# Temporal Variation of *Aristolochia chilensis* Aristolochic Acids during Spring

**DOI:** 10.3390/molecules201119704

**Published:** 2015-11-13

**Authors:** Rocío Santander, Alejandro Urzúa, Ángel Olguín, María Sánchez

**Affiliations:** Facultad de Química y Biología, Universidad de Santiago de Chile, Casilla 40, Correo 33, Santiago 9170022, Chile; angel.olguin@usach.cl (Á.O.); mariapilar.sanchez@uv.cl (M.S.)

**Keywords:** *Aristolochia chilensis*, aristolochic acids, HPLC-DAD, temporal variation

## Abstract

In this communication, we report the springtime variation of the composition of aristolochic acids (AAs) in *Aristolochia chilensis* leaves and stems. The dominant AA in the leaves of all samples, which were collected between October and December, was AA-I (**1**), and its concentration varied between 212.6 ± 3.8 and 145.6 ± 1.2 mg/kg and decreased linearly. This decrease occurred in parallel with the increase in AA-Ia (**5**) concentration from 15.9 ± 0.8 mg/kg at the beginning of October to 96.8 ± 7.8 mg/kg in mid-December. Both acids are enzymatically related by methylation-demethylation reactions. Other AAs also showed important variations: AA-II (**2**) significantly increased in concentration, reaching a maximum in the first two weeks of November and subsequently decreasing in mid-December to approximately the October levels. The principal component in the AA mixture of the stems was also AA-I (**1**); similar to AA-II (**2**), its concentration increased beginning in October, peaked in the second week of November and subsequently decreased. The concentrations of AA-IIIa (**6**) and AA-IVa (**7**) in the leaves and stems varied throughout the study period, but no clear pattern was identified. Based on the variation of AAs in *A. chilensis* leaves and stems during the study period, the reduced contents of non-phenolic AAs and increased concentrations of phenolic AAs are likely associated with a decrease in this plant’s toxicity during the spring.

## 1. Introduction

**Figure 1:**
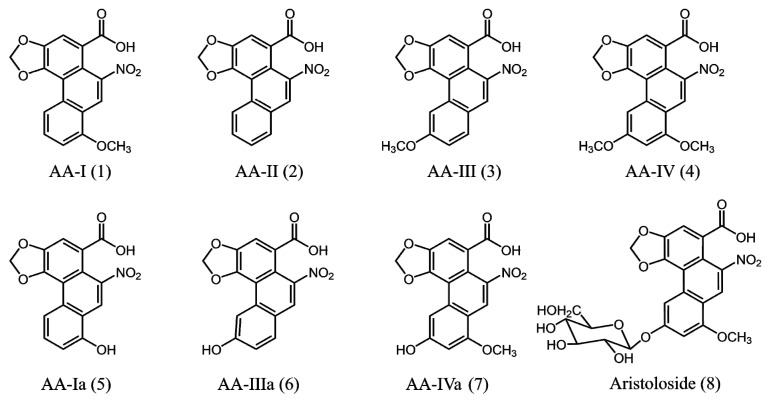
Aristolochic acids in *Aristolochia chilensis*.

Species of the genus *Aristolochia* (Aristolochiaceae) have been used in folk medicine worldwide to treat various diseases [1]. *Aristolochia* contains aristolochic acids (AAs), a group of aporphinoids (10-nitrophenanthrene-1-carboxylic acids). Among the known AAs, AA-I (**1**) and AA-II (**2**) Figure 1, are powerful carcinogens in mice, rats and humans. Studies have shown that these AAs are genotoxic, mutagenic, and nephrotoxic [1,2].

Two species represent the family Aristolochiaceae in Chile: *Aristolochia chilensis* Bridges ex Lindl. and *Aristolochia bridgesii* (Klotzsch) Duch. The former is a summer-deciduous, low-creeping herb that ranges southwards from Caldera in Northern Chile (27°S) to beyond the latitude of Santiago (34°S), and it is known by the local name of “hierba de la Virgen María” (Virgin Mary’s herb) [3]. Teas from the leaves and, particularly, the roots of *Aristolochia chilensis* Bridges ex Lindl. have been used in Central Chile since the 19th century as an anti-hemorrhagic agent and to expel the residual placenta after childbirth [4,5].

In Chilean folk medicine, the common name “Virgin Mary’s herb” of *A. chilensis* is also given to two other medicinal plants (*Stachys albicaulis* Lindl. and *Phyla nodiflora* (L.) Greene). This coincidence of vernacular names has resulted in a consumption of the aerial parts of *A. chilensis* that is much higher than expected because of the confusion of herbalists in rural markets, who may have no botanical training [6,7]. Although the risk of using *A. chilensis* in folk medicine is evident, the plant continues to be used. 

A recent study has shown that the roots of *Aristolochia chilensis* contain a mixture of AA-I (**1**), AA-II (**2**), AA-III (**3**), AA-IV (**4**), AA-Ia (**5**), AA-IIIa (**6**), AA-IVa (**7**), and aristoloside (**8**) (a 6-*O*-β-d-glucopyranoside of AA-IVa (**7**)). This mixture is rich in phenolic AAs and AA-IVa (**7**), and aristoloside (**8**) accounts for approximately 90% of the total AAs. In contrast, the most toxic compound, AA-I (**1**), is a minor component [8]. Previous investigations of the aerial parts of *A. chilensis* revealed the presence of a mixture of AA-I (**1**), AA-II (**2**), AA-Ia (**5**), AA-IIIa (**6**) and AA-IVa (**7**) and trace amounts of AA-III (**3**) and AA-IV (**4**) [9]. In contrast to the roots, the main AA component of the aerial parts of *A. chilensis* is AA-I (**1**), which accounts for approximately 50% of the total AAs [8]. Because the most toxic AA is AA-I (**1**) [10], human consumption of the aerial parts of *A. chilensis* is likely more dangerous than the consumption of the root.

*Aristolochia chilensis* blooms from mid-August to early September, reaches the peak of vegetative development in late November, and declines during December, at which time, the aerial parts are lost. Because of the increasing concern caused by the indiscriminate consumption of *A. chilensis*, we evaluated the variation in the AA content and composition in the leaves and stems of a population of this plant during harvesting by herbalists. In this study, high-performance liquid chromatography with diode-array detection (HPLC-DAD), which is a widely used technique to detect and quantify AAs in herbal medicine [8,9,11,12,13,14,15,16], was used to determine the amounts of AA-I (**1**), AA-II (**2**), AA-Ia (**5**), AA-IIIa (**6**) and AA-IVa (**7**) from samples of *A. chilensis* leaves and stems, which were collected during spring.

## 2. Results and Discussion

We determined the contents of AA-I (**1**), AA-II (**2**), AA-Ia (**5**), AA-IIIa (**6**) and AA-IVa (**7**) from samples of *A. chilensis* leaves and stems, which were collected in spring between October and December. The results are summarized in Table 1 and Table 2.

**Figure 2:**
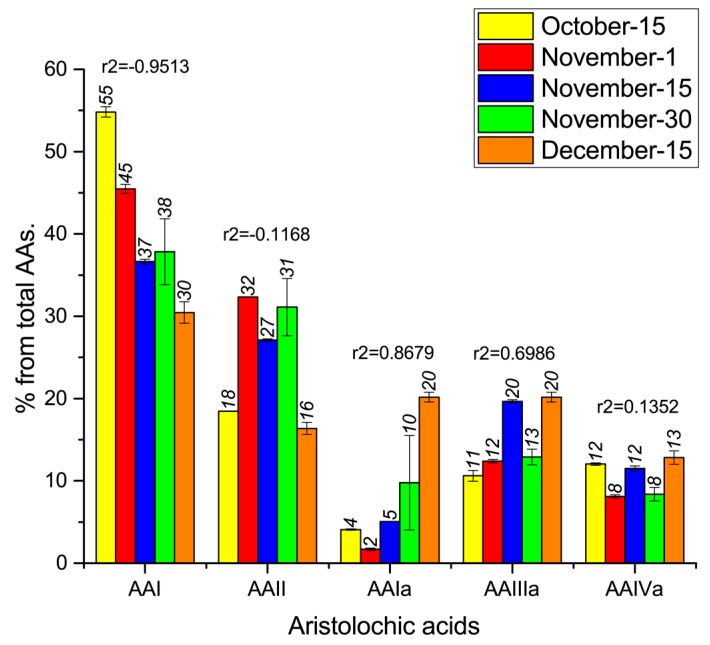
Variation during springtime of the percentage (%) of each aristolochic acid (AA), in the total AAs mixture from leaves samples.

The dominant AA in the leaves of all collected samples between October and December was AA-I (**1**); AA-I(**1**) clearly decreases of 25% but still his content is 30% of the total AAs in December, Pearson coefficient (r2 −0.9513), whereas AA-Ia (**5**) increases linearly of 20% and showed a positive Pearson coefficient (r2 0.8679), Figure 2.

This increase occurred in parallel with the decrease in the AA-I (**1**) concentration. Because AA-I (**1**) and AA-Ia (**5**) are related by simple enzymatic methylation-demethylation reactions, the decrease in the AA-I (**1**) concentration is directly related to the increase in the AA-Ia (**5**) concentration. In addition, AA-II (**2**) can be hydroxylated to AA-Ia (**5**) and AA-IIIa (**6**), also shows a weak positive Pearson coefficient (r2 0.6986). AA-I (**1**) has been found to be severely cytotoxic and elicits maximal toxicity in renal epithelial cells (IC_50_ value: 10 mmol/L), whereas AA-Ia (**5**) is twenty times less toxic (IC_50_ value: 200 mmol/L) [10].

No correlation was found in the variation of AA-II (**2**) and AA-IVa (**7**) (Figure 2). The AA contents in the stems of the collected samples between October and December showed no normality (Shapiro-Wilk test). However, from October to December, the concentrations of AA-IIIa (**6**) and AA-IVa (**7**) increased by 7.4 and 16.8 times, respectively. Additionally, AA-Ia (**5**) was detected in the November and December samples (Table 2).

**Table 1:** Content (mg/kg, X ± SD) of AA-I (**1**), AA-II (**2**), AA-Ia (**3**), AA-IIIa (**4**) and AA-IVa (**5**) from samples of leaves of *A. chilensis* collected between October and December 2014.

	Non Phenolic Acids		Phenolic Acids			
*Harvest Date*	*AA-I*	*AA-II*	*AA-Ia*	*AA-IIIa*	*AA-IVa*	*Total AAs*
October-15	212.6 ± 3.8	71.6 ± 2.2	15.9 ± 0.8	41.2 ± 3.7	46.7 ± 0.8	388.0 ± 5.9
November-1	206.3 ± 5.8	146.8 ± 5.9	7.7 ± 1.0	56.3 ± 3.2	36.9 ± 2.4	453.9 ± 9.2
November-15	195.5 ± 2.8	144.6 ± 2.4	26.9 ± 0.3	104.9 ± 3.2	61.5 ± 2.8	533.5 ± 5.7
November-30	154.5 ± 0.8	129.7 ± 3.1	61.9 ± 8.1	49.4 ± 9.5	31.4 ± 4.7	426.9 ± 13.7
December-15	145.6 ± 1.2	78.3 ± 0.5	96.8 ± 7.8	96.8 ± 7.8	61.6 ± 7.1	479.1 ± 13.2

**Table 2:** Content (mg/kg, X ± SD) of AA-I (**1**), AA-II (**2**), AA-Ia (**3**), AA-IIIa (**4**) and AA-IVa (**5**) from samples of stems of *A. chilensis* collected between October and December 2014.

	Non Phenolic Acids		Phenolic Acids			
*Harvest Date*	*AA-I*	*AA-II*	*AA-Ia*	*AA-IIIa*	*AA-IVa*	*Total-AAs*
October-15	104.6 ± 3.2	20.6 ± 0.7	-	12.3 ± 2.5	5.4 ± 1.0	142.8 ± 4.2
November-1	307.5 ± 3.0	68.4 ± 1.5	-	39.2 ± 2.0	23.7 ± 2.2	438.8 ± 4.5
November-15	325.3 ± 8.2	118.6 ±3.7	-	49.3 ± 2.0	47.1 ± 2.9	540.3 ± 9.7
November-30	294.6±10.6	102.1 ±2.9	8.8 ± 0.5	41.2 ± 3.0	39.4 ± 3.0	486.1 ± 11.8
December-15	265.4 ± 3.7	65.5 ± 3.4	46.9 ± 3.6	90.6 ± 9.2	90.6 ± 9.8	559.0 ± 14.8

## 3. Experimental Section

### 3.1. Plant Material

Fieldwork was performed at Cuesta Lo Prado (15 km west of Santiago, 33°28′S, 70°56′W, 750 m above sea level) from mid-October to mid-December. Thirty *Aristolochia chilensis* plants of similar sizes and phenological stages were chosen and marked (N° 1–30). From this group, 5 plants were randomly selected every fifteen days. Leaf and stem samples of each of the five plants were collected and immediately oven-dried at 50 °C for 24 h, milled and subjected to extraction and analysis. 

### 3.2. Extract Preparation

Each oven-dried and powdered sample (20 g) was extracted with methanol (160 mL) at room temperature for 24 h and subsequently boiled for 4 h. The suspension was vacuum filtered, and the plant material was washed with methanol (40 mL). The combined extract was evaporated *in vacuo*.

The syrupy residue was agitated with 60 mL of 5% NaHCO_3_ at 40 °C, allowed to stand at room temperature and washed with CHCl_3_ (4 × 15 mL) and ethyl acetate (4 × 15 mL). Washing with CHCl_3_ and AcOEt after evaporation yielded brown or dark-green extracts that contained no acids, which were not investigated further. The aqueous phase was adjusted to pH 2 using HCl and extracted with ethyl acetate (4 × 15 mL). Evaporation of the combined extracts *in vacuo* yielded a fraction of crude AAs. The procedure was repeated for 25 samples (20 g) of leaves and 25 (20 g) samples of stems, which were obtained from five different plants on each collection date.

### 3.3. HPLC Analysis of AAs 

**Figure 3:**
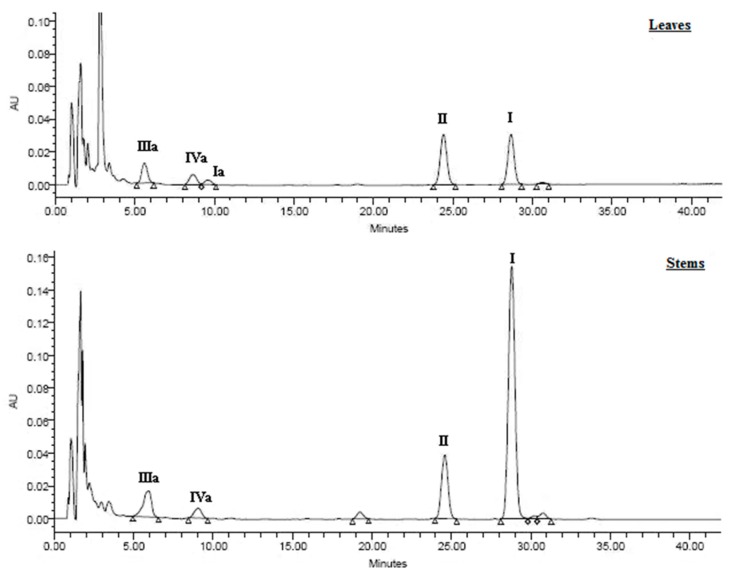
Representative chromatograms of leaves and stems aristolochic acids from *Aristolochia chilensis*.

The crude AA fractions were analyzed using HPLC (Waters 600; Milford, MA, USA) with a reverse-phase Symmetry Shield RP18 column (5-μm particle size; 25 × 0.46 cm). The method was described in a previous paper [16]. Briefly, gradient elution was performed as follows using a mobile phase, which consisted of 0.1% acetic acid in water (solution A) and 0.1% acetic acid in acetonitrile (solution B): 0–5 min, isocratic elution with 70% A/30% B; 5–45 min, linear gradient from 70% A/30% B to 55% A/45% B. A Waters 2996 diode-array-detector (DAD) was used to detect the AAs, and their spectra were recorded at wavelengths of 200–800 nm. The UV spectra and retention times of all detected AAs were coincident with the AA-I (**1**), AA-II (**2**), AA-Ia (**5**), AA-IIIa (**6**) and AA-IVa (**7**) standards, Figure 3 [8,9]. Quantification was based on the areas of the peaks in the chromatograms, which were determined at 254 nm. A dilution series of standard solutions was prepared from stock solutions of the standards and all standard and sample solutions were stored at 5 °C. Calibration curves were obtained by plotting the peak areas against the standard concentrations; these curves were used to determine the AA concentrations in the samples.

### 3.4. Statistical Analysis

The Shapiro-Wilk test was used in data normality testing. Linear correlations between the concentrations of AAs and the dates when the plant samples were collected were assessed by calculating the Pearson correlation coefficient. 

## 4. Conclusions

The findings reveal that the AA composition of *Aristolochia chilensis* leaves changes throughout spring, exhibiting a linear decrease in the concentration of the non-phenolic acid AA-I (**1**) and linear increases in the concentration of the phenolic acids AA-Ia (**5**) and AA-IIIa (**6**). The variations of AA-II (**2**) and AA-IVa (**7**) were not adjusted to a linear correlation model. However, from October to December, the total concentration of phenolic AAs increased, and the concentration of non-phenolic AAs decreased. The AA concentrations in the stems collected between October and December showed no normality (Shapiro-Wilk test). Based on the variation of the AAs in *A. chilensis* leaves and stems during the study period, the reduced content of non-phenolic AAs and the increased concentration of phenolic AAs are likely associated with a decrease of this plant’s toxicity during the spring.
